# Direct-acting antiviral treatment downregulates immune checkpoint inhibitor expression in patients with chronic hepatitis C

**DOI:** 10.1007/s10238-020-00618-3

**Published:** 2020-02-27

**Authors:** Laszlo Szereday, Matyas Meggyes, Timea Berki, Attila Miseta, Nelli Farkas, Judit Gervain, Alajos Par, Gabriella Par

**Affiliations:** 1 Department of Medical Microbiology and Immunology, University of Pecs, Medical School, 12 Szigeti Street, Pecs, 7624 Hungary; 2 Department of Biotechnology and Immunology, University of Pecs, Medical School, Pecs, Hungary; 3 Department of Laboratory Medicine, University of Pecs, Medical School, Pecs, Hungary; 4 Institute for Translational Medicine, University of Pecs, Medical School, Pecs, Hungary; 5 County Hospital Fejér, Szent György Hospital, Szekesfehervar, Hungary; 6 Division of Gastroenterology, First Department of Medicine, University of Pecs, Medical School, Pecs, Hungary; 7 Janos Szentagothai Research Centre, Pecs, Hungary

**Keywords:** Hepatitis C, Direct-acting antivirals, Checkpoint inhibitors, TIM-3/galectin-9 pathway, PD-1/PD-L1 pathway

## Abstract

Chronic hepatitis C (CHC) infection is associated with increased TIM-3, PD-1 immune checkpoint receptors expression that inhibits adaptive T cells and increases NK cell cytotoxicity against T helper cells, both resulting T cell exhaustion. Elimination of the virus with direct-acting antivirals (DAAs) may modify host immune response via altering these immune checkpoint receptors’ expression. We conducted a prospective study to analyze changes in TIM-3, PD-1 and their ligands galectin-9, PD-L1 expression by peripheral blood T cell subpopulations, NK cell subpopulations, and monocytes by multicolor flow cytometry in 14 CHC patients successfully treated with 12 weeks of dasabuvir, ombitasvir, and paritaprevir/ritonavir plus ribavirin. Blood samples were collected before, at the end of treatment, and 12 and 24 weeks later. Sustained virological response (SVR) was associated with increased percentage of peripheral blood CD3+ T and CD8+ cytotoxic T lymphocytes and decreased percentage of NKbright cells. After DAA treatment, decreased TIM-3 expression by CD4+ T cells, by NKbright, and by NKT cells was found. Expression of immune checkpoint molecules’ ligand PD-L1 by NK cells and by regulatory T cells and galectin-9 by NK cells and monocytes also decreased significantly at SVR. Our data suggest that DAA treatment not only inhibits viral replication but may alter host adaptive and innate immune responses. A decrease in immune checkpoint molecules and their ligands expression both on adaptive and on innate immune cells may contribute to the recovery of exhausted adaptive immune responses and to sustained virological response.

**Electronic supplementary material:**

The online version of this article (10.1007/s10238-020-00618-3) contains supplementary material, which is available to authorized users.

## Introduction

In chronic hepatitis C virus (HCV) infection, the constant presence of the viral antigens results in exhausted adaptive T cell responses. HCV-specific CD4+ T cells are weakened and burdened with a decrease in IL-2 secretion capacity [1], and HCV-specific CD8+ T cells have impaired effector function associated with decreased antiviral cytokine (IL-2, TNF, and IFN-γ) production [2–4]. Immune checkpoint inhibitor molecules are important regulators of immune response during HCV infections. Virus-specific CD8 T cell exhaustion is associated with sustained upregulation of co-inhibitory checkpoint molecules, including PD-1, TIM-3 [5]. Dysregulation of TIM-3 expression on NK cells has been described as a characteristic feature regarding several chronic viral infections [6, 7]. In contrast to T cells, high levels of TIM-3 expression on NK cells are associated with an activated phenotype toward cytotoxicity. Tim-3+ NK cells cytolytically eliminate activated CD4 T cells that affect CD8 T cell function and contribute to immune dysfunction and viral persistence via restricting the induction of adaptive anti-viral T cell responses [8].

Viral infection enhances TIM-3 expression indirectly through the induction of pro-inflammatory cytokines, such as IL-12 or IL-15 produced by dendritic cells or activated monocytes/macrophages. Elevated PD-1’s ligand PD-L1 expression by hepatocytes also contributes to reduced T cell function [9]. Furthermore, expression of galectin-9 (Gal-9), the ligand for TIM-3, is also upregulated by Kupffer cells and monocytes in HCV and induces the expansion of CD4+ regulatory T cells (Treg) in a TGF-β-dependent manner and apoptosis of HCV-specific cytotoxic lymphocytes (CTLs) [10, 11].

While the persistently high levels of the virus in the host drive the exhaustion regarding the T cell response, the viral level drop during the spontaneous resolution of HCV infection results in regained functionality of anti-viral CD8+ T cells [12]. Advances in the development of combination therapy of HCV-specific directly acting antivirals (DAAs) revolutionized HCV therapy [13]. DAAs with very high cure rates suppress HCV replication and terminate chronic antigen stimulation and, consequently, may act upon the host’s immune responses [14]. During DAA-mediated HCV clearance, HCV-specific CD8+ T cells proliferative capacity improve along with augmented IFN-γ responses and regain antiviral effector functions [15]. A rapid decrease in viremia and the decreased level of inflammatory cytokines are also associated with the reduced activation of intrahepatic and blood innate immune cells, followed by the restoration of a normal NK cell phenotype and function [16]. The exact immunomodulatory role of DAA is not known; however, there have been several unexpected sequelae reported suggesting DAAs potential effect on host innate immune response such as increased rate of reactivations of hepatitis B virus (HBV) or herpesviruses, higher occurrence of de novo or provoked autoimmune diseases, or conflicting results regarding high early recurrence rates of hepatocellular carcinoma (HCC) [17–21]. In the present prospective study, we investigated the phenotypic changes and the expression of different inhibitory immune checkpoint receptors (PD-1, TIM-3) and their ligands (PD-L1, Gal-9) by peripheral blood mononuclear cell subpopulations before and after 12 and 24 weeks of dasabuvir, ombitasvir, and paritaprevir/ritonavir plus ribavirin combination treatment. We found that DAA-induced sustained virological response (SVR) was associated with decreased TIM-3 immune checkpoint receptor and decreased PD-L1 and galectin-9 ligand expression. Since activated TIM-3+ NK cells play a vital role as a rheostat in controlling T cells by direct T cell killing or production of regulating cytokines, the decrease IN the percentage of TIM3+ NK cells may favor the re-establishment of T cell responses.

## Materials and methods

### Patients and samples

Heparinized venous blood samples were collected from 14 patients afflicted with chronic hepatitis C infection, all of whom were additionally suffering from compensated liver disease. All Patients were diagnosed with a HCV1b genotype infection and were HBV and HIV seronegative. The characteristics of these patients are presented in Table 1.

**Table 1:** Patient characteristics of the study cohort at baseline

Parameter	Baseline (*N *= 14)
Age (years)	68 (54–78)
Male sex (%)	47% female, 53% male
BMI (kg/m^2^)	27 (24–33)
RNA IU/ml	5.34 × 10^5^ (3.75 × 10^4^–2.48 × 10^6^)
Genotype (%)	
1b	100% (14)
ALT (U/I)	76 (21–194)
AST (U/I)	84 (21–151)
Albumin (g/l)	44 (32–47)
Platelets (× 10^3^/µl)	158 (39–289)
APRI	1.192 (0.165–5.886)
FIB-4 Index	2.975 (0.77–18.48)
Liver cirrhosis—no/total no (%)	12/14 (85.7%)
LS (kPa)	20.4 (7.8–35.3)

Values are given as median and range

*BMI* body mass index, *ALT* alanine aminotransferase, *AST* aspartate aminotransferase, *APRI* AST to platelet ratio index, *FIB-4 Index* Fibrosis-4 Index, *F* fibrosis, *LS* liver stiffness, *kPa* kilopascal

Patients were treated for a span lasting 12 weeks, including dasabuvir, ombitasvir, and paritaprevir/ritonavir plus ribavirin combination treatment, the only available and reimbursed DAA treatment in Hungary at the time of patient inclusion (2017). All patients achieved sustained virological response. Samples were collected at baseline, at the end of treatment (EOT), 12 (SVR12), and 24 weeks following EOT (SVR24).

### Lymphocyte separation, cryopreservation, and thawing

Peripheral blood mononuclear cells (PBMC) were separated on the Ficoll–Paque density gradient. The collected cells were then washed in RPMI 1640 medium, counted, centrifuged, and resuspended in human serum containing 10%DMSO for cryoprotection. Subsequently, cells were aliquoted in cryovials and stored in a − 80 °C mechanical freezer. Thawing was carried out on the day of fluorescent cell labeling as quickly as possible in a 37 °C water bath, and DMSO underwent two rinse cycles in RPMI 1640 medium. The viability of the cells was assessed utilizing trypan blue exclusion (consistently > 90%).

### Flow cytometry analysis

#### Antibodies

Freshly thawed PBMC were used for surface and intracellular staining regarding flow cytometric analysis. The following monoclonal antibodies were used: Brilliant Violet (BV421)-conjugated anti-human PD-L1 (BD Biosciences), BV510-conjugated anti-human CD3 (BD Biosciences), fluorescein isothiocyanate (FITC)-conjugated anti-human CD4 (BD Biosciences), FITC-conjugated anti-human CD14 (BD Biosciences), (phycoerythrin (PE)-conjugated anti-human PD-1 (Beckmann-Coulter), PE-conjugated anti-human Gal-9 (Biolegend), Peridinin Chlorophyll Protein (PerCP)-conjugated anti-human CD56 (BD Biosciences), PerCP-conjugated anti-human Perforin (BD Biosciences), allophycocyanin (APC)-conjugated anti-human TIM-3 (R&D Systems), APC-conjugated anti-human CD56 (BD Biosciences), APC-conjugated anti-human FoxP3 (eBioscience), APC/H7-conjugated anti-human CD8 (BD Biosciences).

#### Lymphocyte labeling and flow cytometric measuring

Cell surface expression among a varied immune subpopulation was analyzed using fluorochrome-conjugated monoclonal antibodies. One hundred and six cells in 100 µl phosphate-buffered saline (PBS)/tube were incubated for 30 min at room temperature including the fluorochrome-labeled monoclonal antibodies. Following the staining, cells were rinsed in PBS, fixed in 300 µl PBS containing 1% paraformaldehyde (PFA), and stored at 4 °C in complete darkness until FACS analysis. Data acquisition and analyses were performed using the FACS Canto flow cytometer (BD Biosciences, San Diego, CA, USA) equipped with the FACSDIVA V6. software program (BD Biosciences, San Diego, CA, USA).

#### Intracellular perforin staining

Following surface labeling, cells were next rinsed and fixed in 4% PFA for 10 min at room temperature. Additionally, the cells were rinsed twice using PBS and incubated with 1:10 diluted FACS Permeabilizing Solution 2 (BD Biosciences) for 10 min at room temperature and then rinsed twice using PBS. Permeabilized cells were then incubated with PerCP-conjugated anti-human perforin for 30 min at room temperature, in complete darkness. The samples then rinsed using PBS, fixed with PBS containing 1% PFA, and stored at 4 °C in complete darkness until the FACS analysis.

#### Staining of Treg cells

Following the surface staining with anti-CD3 and anti-CD4 antibodies, intracellular labeling of FoxP3 was performed using the FoxP3 Staining Buffer Set (eBioscience) in accordance with the manufacturer’s protocol. Briefly, cells were permeabilized in 1 ml fixation/permeabilization buffer (concentrate/diluent 1:4) at 4 °C for 1 h in complete darkness. Next, the samples were rinsed twice in the buffer and stained with the APC-conjugated anti-human FoxP3 monoclonal antibody at 4 °C for 1 h in complete darkness. The samples then were rinsed twice using PBS, fixed with PBS containing 1% PFA and stored at 4 °C in complete darkness until the FACS analysis.

### Statistical analysis

Statistical analysis was performed using statistical software SPSS version 20 Package. Significant differences were calculated with parametric repeated-measures ANOVA in association with the Bonferroni correction and nonparametric Friedman test. Differences were considered significant if the *p* value was equal to or less than 0.05.

## Results

### DAA treatment resulted in an increased percentage of peripheral blood CD3+ and CD8+ T cells and a decreased percentage of NKbright cells

**Fig. 1:**
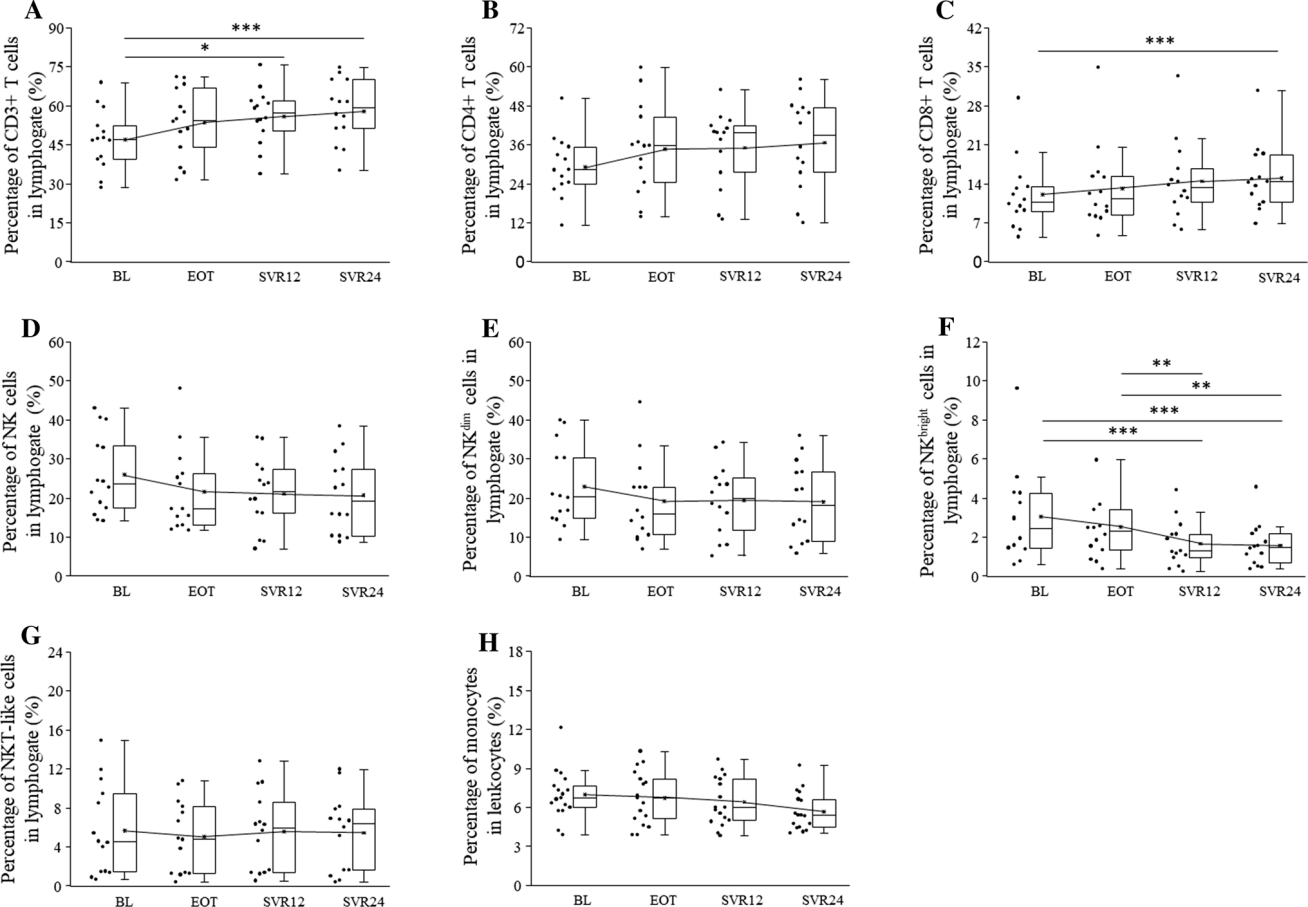
Peripheral blood mononuclear cells characteristics in HCV patients on DAA treatment. Distribution of CD3+ T, CD4+ T, CD8+ T, NK, NKdim, NKbright, NKT-like, and monocytes in HCV patients on DAA treatment. The solid bars represent medians of 14 determinations, and the boxes indicate the interquartile ranges and the lines show the most extreme observations. Differences were considered statistically significant for *p* values ≤ 0.05

Peripheral blood mononuclear cells were examined among HCV patients, at the end and 12 and 24 weeks following the conclusion of DAA treatment (SVR12 and SVR24) via multicolor flow cytometry (Fig. 1 and Supplementary Table 1).

The percentage of CD3+ T increased over time, and there were significant differences between BL and SVR12 and between BL and SVR24 (Fig. 1a). The percentage of CD8+ T cells also increased over time, and there was a significant difference between BL and SVR24 (Fig. 1c). The percentage of NKbright cells decreased over time, and there were significant differences between BL and SVR12, between BL and SVR24, between EOT and SVR12 and between EOT and SVR24 (Fig. 1f).

The frequency of CD4+ T, NK, NKdim, NKT-like, Treg cells, and monocytes did not differ over the entire observation period (Fig. 1b, d, e, g, h and Supplementary Table 1).

### DAA treatment decreased TIM-3 and PD-1 immune checkpoint receptor expression by peripheral blood CD4+ T cells and by NK or NKT-like cells

We measured the surface expression of TIM-3 and PD-1 by CD3+, CD4+, CD8+ T cells, NK, NKdim, NKbright, and NKT-like cells with flow cytometry.

**Fig. 2:**
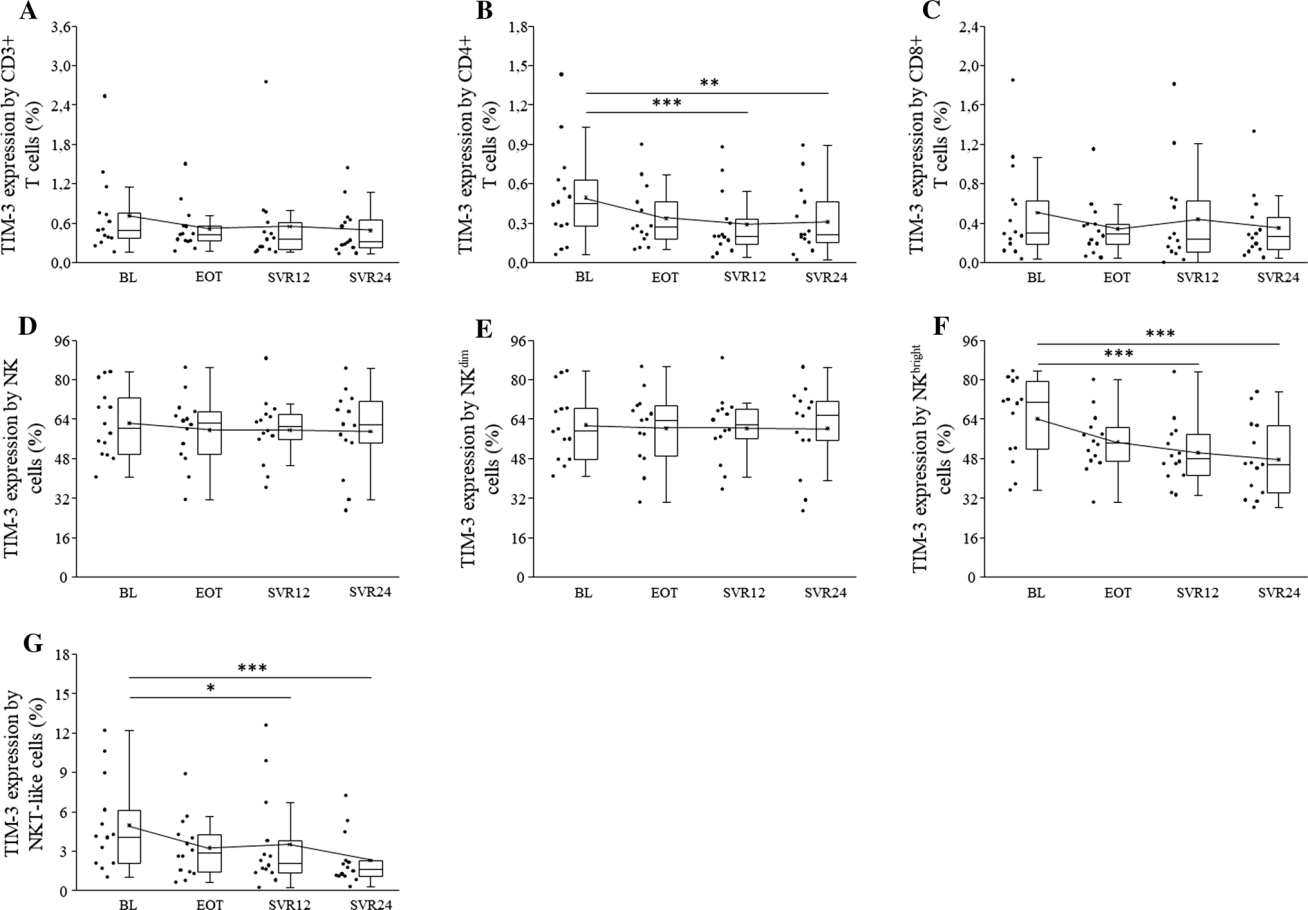
TIM-3 expression by peripheral blood mononuclear cells in HCV patients on DAA treatment. The expression of TIM-3 by CD3+, CD4+, CD8+ T cells, NK, NKdim, NKbright and NKT-like cells in HCV patients on DAA treatment. The solid bars represent medians of 14 determinations, the boxes indicate the interquartile ranges and the lines show the most extreme observations. Differences were considered statistically significant for *p* values ≤ 0.05

The percentage of TIM-3 positive CD4+, NKbright, and NKT-like cells decreased over time, and there were significant differences between BL and SVR12 and between BL and SVR24 (Fig. 2b, f, g).

**Fig. 3:**
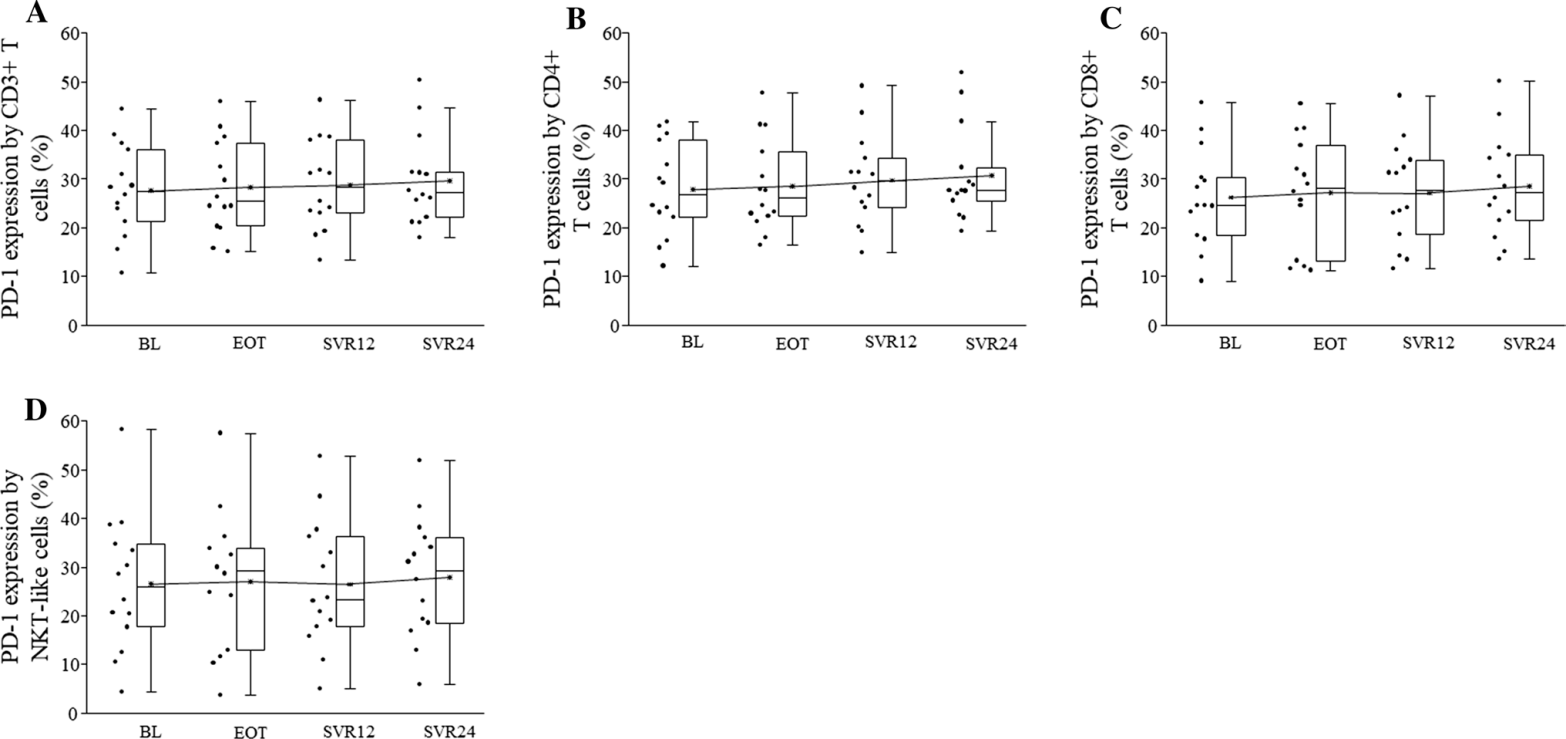
PD-1 expression by peripheral blood mononuclear cells in HCV patients on DAA treatment. The expression of PD-1 by CD3+ T, CD4+, CD8+ T cells, and NKT-like cells in HCV patients on DAA treatment. The solid bars represent medians of 14 determinations, the boxes indicate the interquartile ranges and the lines show the most extreme observations. Differences were considered statistically significant for *p* values ≤ 0.05

The percentage of TIM-3 positive CD3+ T, CD8+ T, NK, and NKdim cells did not differ regarding the patients spanning the entire observation period (Fig. 2a, c, d, e). PD-1 was undetectable in NK, NKdim, and NKbright cells and PD-1 expression did not change significantly by CD3+, CD4+, CD8+ T, and NKT-like cells during DAA treatment (Fig. 3a–d).

### SVR was associated with decreased PD-L1 and galectin-9 immune checkpoint ligand expression by NK cells

**Fig. 4:**
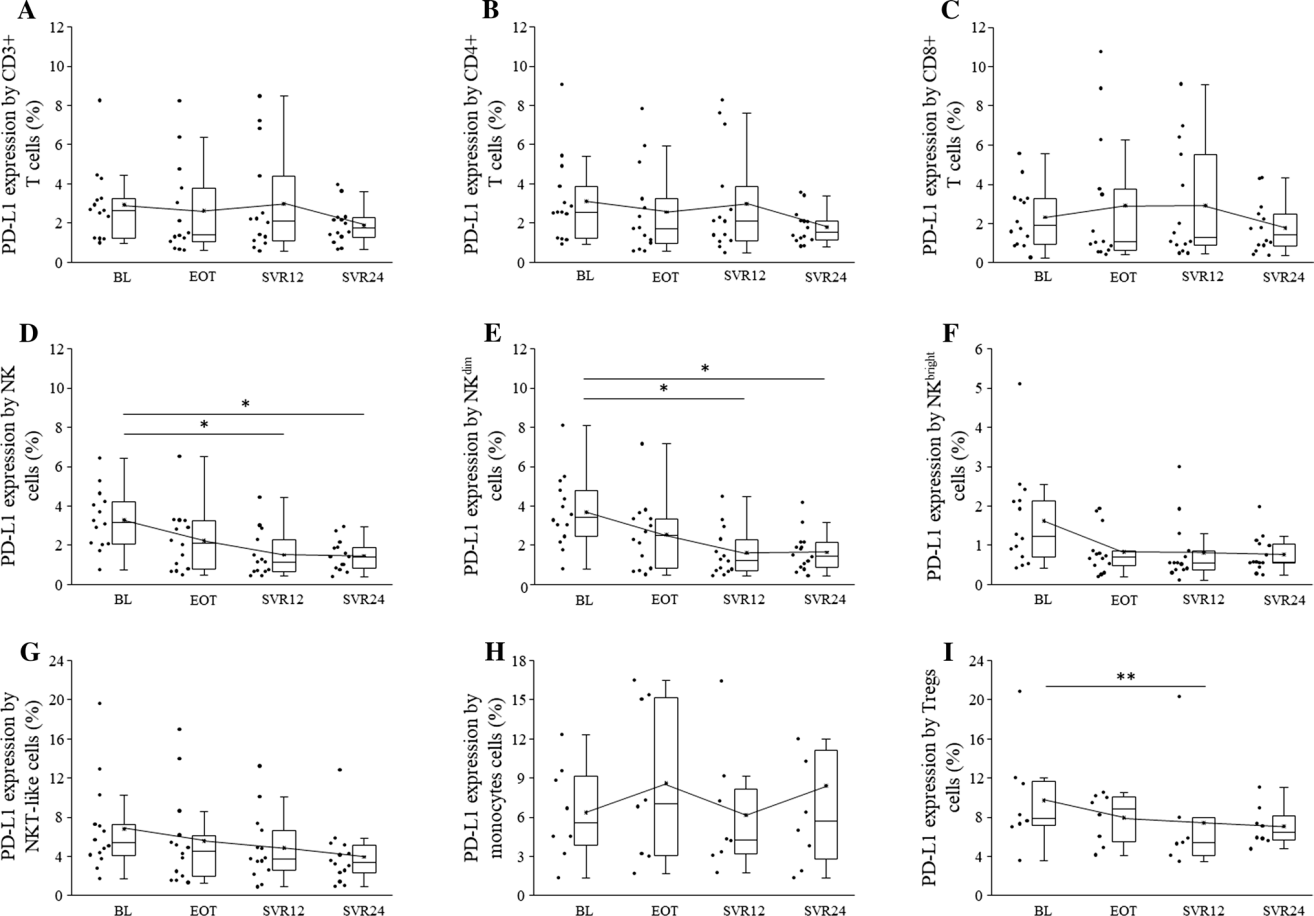
PD-L1 expression by peripheral blood mononuclear cells in HCV patients on DAA treatment. The expression of PD-L1 by CD3+, CD4+, CD8+ T cells, NK, NKdim, NKbright and NKT-like cells, monocytes and Treg cells in HCV patients on DAA treatment. The solid bars represent medians of 14 determinations, and the boxes indicate the interquartile ranges and the lines show the most extreme observations. Differences were considered statistically significant for *p* values ≤ 0.05

The percentage of PD-L1 positive NK and NKdim cells decreased over time, and there were significant differences between BL and SVR12 and between BL and SVR24 (Fig. 4d, e). The percentage of PD-L1 positive Treg cells also decreased between BL and SVR12 (Fig. 4i).

**Fig. 5:**
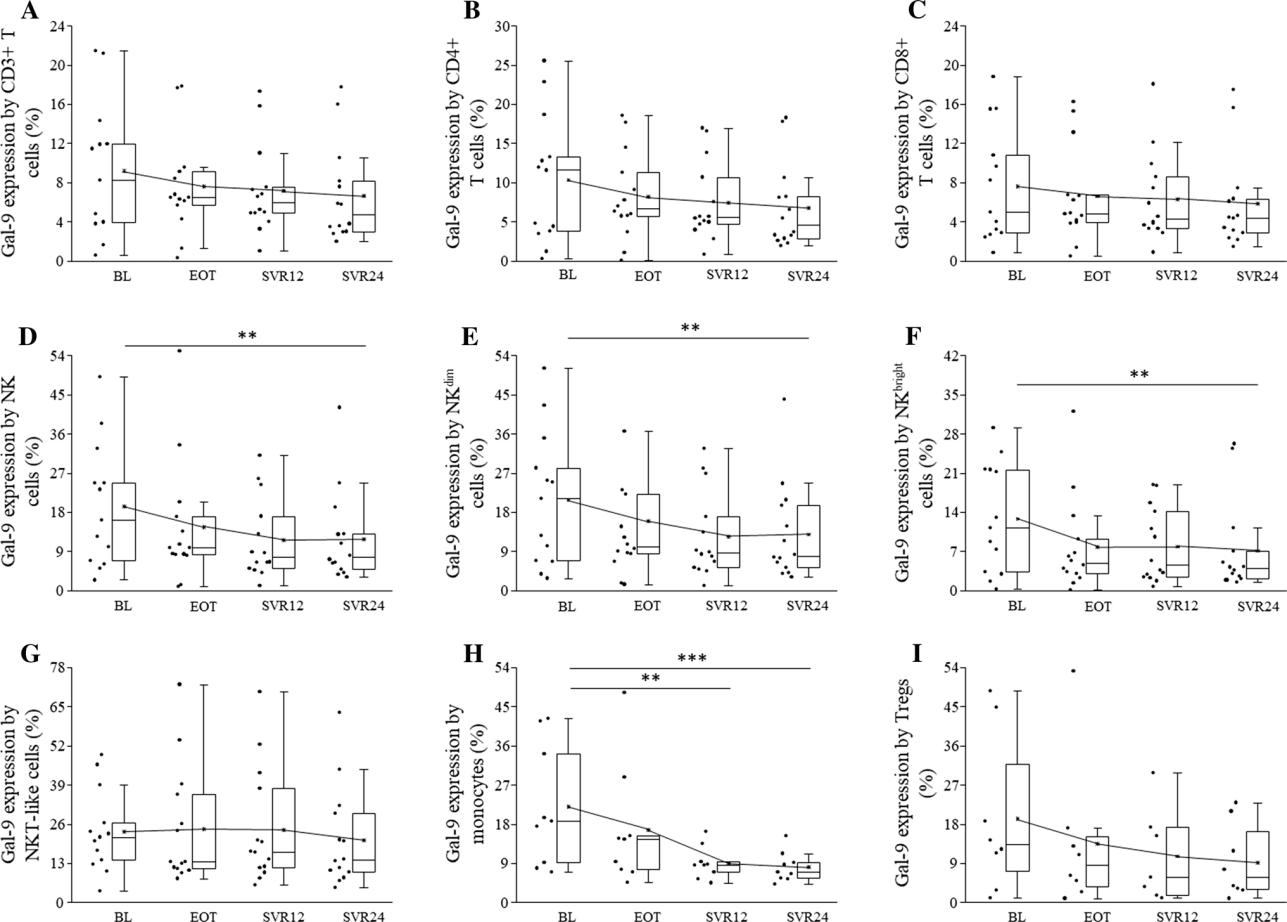
Gal-9 expression by peripheral blood mononuclear cells in HCV patients on DAA treatment. The expression of PD-L1 by CD3+, CD4+, CD8+ T cells, NK, NKdim, NKbright and NKT-like cells, monocytes and Treg cells in HCV patients on DAA treatment. The solid bars represent medians of 14 determinations, and the boxes indicate the interquartile ranges and the lines show the most extreme observations. Differences were considered statistically significant for *p* values ≤ 0.05

The percentage of Gal-9 expressing NK, NKdim, and NKbright cells decreased over time, and there was a significant difference between BL and SVR24 (Fig. 5d, e, f). Significantly decreased Gal-9 expression by monocytes was also observed at SVR12 and SVR24 compared to BL (Fig. 5h).

PD-L1 expression by CD3+, CD4+, CD8+ T, NKbright, NKT-like cells, and monocytes did not show any significant difference over time (Fig. 4a–c, f–h). Gal-9 expression by CD3+, CD4+, CD8+ T, NKT-like, and Tregs did not change significantly over time (Fig. 5a–c, g, i).

### Increased perforin expression by peripheral blood NK cells in HCV patients at SVR24

**Fig. 6:**
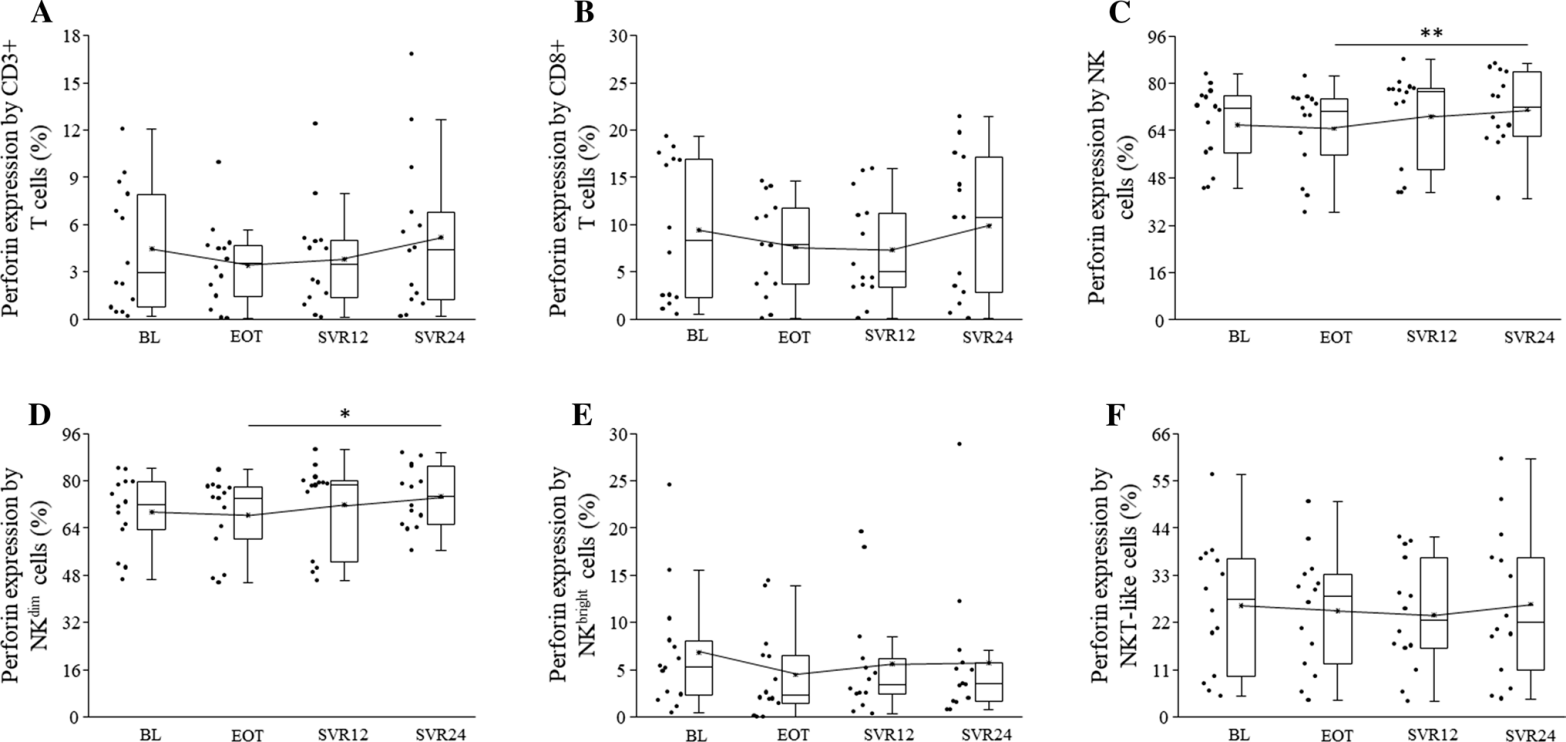
Intracellular perforin expression by peripheral blood mononuclear cells in HCV patients on DAA treatment. The expression of intracellular perforin by CD3+, CD8+ T cells, NK, NKdim, NKbright and NKT-like cells in HCV patients on DAA treatment. The solid bars represent medians of 14 determinations, and the boxes indicate the interquartile ranges and the lines show the most extreme observations. Differences were considered statistically significant for *p* values ≤ 0.05

The intracellular perforin content significantly increased at SVR24 in NK and NKdim cells compared to EOT (Fig. 6c, d). DAA treatment did not change CD3+, CD8+, NKbright, and NKT-like cells perforin expression (Figure 6a, b, e, f).

## Discussion

### The role of immune checkpoint molecules in HCV infection and the effects of DAA treatment

Chronic HCV infection is associated with impaired CD4+ helper T cell function, exhaustion of CD8+ cytotoxic T cells with suppressed IL-2, TNF, and IFN-γ secretion [22]. Elimination of the hepatitis C virus by DAA treatment may modify the altered host immune response, so our study focused on the analysis of phenotype distribution and different immune checkpoint molecules and their ligands expression by PBMC during DAA therapy.

Several mechanisms have been suggested to contribute immune dysfunction in CHC, such as the immunoregulatory properties of HCV proteins, the availability of CD4 T cell help, and the increased number of CD4+ CD25+ Treg cells [22]. The quality and character of antigen-presenting cell (APC) population, the strength of the cytotoxic natural killer cell response, the composition of cytokine milieu and also the increased expression of different immune checkpoint molecules or immune checkpoint molecule ligands which prevent continuous T cell stimulation, uncontrolled T cell responses, and consequential immune-mediated tissue damage also play a role in lymphocytes nonfunctionality [4].

In chronic HCV infection, the increased expression of checkpoint inhibitors such as TIM-3, LAG3, and TIGIT and their association of exhaustion of cytotoxic T cells have been described [23]. HCV infection may enhance TIM-3 expression directly by HCV proteins or indirectly through induction of IL-12 or IL-15 production by dendritic cells or activated monocytes [9]. HCV peptide-stimulated CD4+ T cells of CHC patients displayed relatively high levels of expression of CTLA-4, PD-1 and TIM-3 [24–26]. TIM-3 is also upregulated on HCV peptide-stimulated CD8+ T cells [5, 24] and on PD-1+ HCV-specific CTLs. TIM-3 blockade restores hepatocyte-directed *in vitro* cytotoxicity [27]. Liver-derived exhausted CD8 T cells from chronically infected HCV patients co-express CTLA-4 and PD-1, and the only blockade of both pathways restores their functionality [28]. The suppression of HCV-specific lymphocyte proliferation, differentiation, cytokine secretion is also regulated by PD-1/PD-L1 pathway by PD-1 upregulation on peripheral and liver-infiltrating Treg cells. HCV-infected human hepatocytes upregulate TIM-3 expression and drive CD4+ T cells to CD25+ Fox3+ Treg cells [29]. HCV patients have a higher number of Tregs when compared with healthy individuals, and the depletion of CD4+ CD25+ T cells enhances antigen-specific CD4+ and CD8+ T cell proliferation and responses.

Immune checkpoint molecules play a critical role in regulating the activities of innate immune cells, such as monocytes, dendritic cells, and NK cells. NK cells destroy not only virus-infected cells but immune cells of the adaptive immune response or produce cytokines that modulate adaptive immunity and directly inhibit virus replication. NK cells in chronic HCV Hepatitis are characterized by a functional dichotomy, featuring enhanced cytotoxicity and reduced production of IFN-γ and TNF-α caused by altered IFN-α signaling. NK cells exhibit a polarized phenotype through TRAIL expression, which may contribute to liver damage and cytotoxicity [30–33]. NK cell activity is regulated by a complex network consisting of activating and inhibiting receptors. The expression of NK cell inhibitory receptors correlates with virus control and decreased liver damage in human cohorts [34]. The PD-1 expression on mature CD56dimCD57+ cells results in poor cytokine production and cytolytic activity compared to PD-1 negative mature NK cells [35]. TIM-3 protein is a maturation marker expressed on essentially all mature CD56dimCD16+ NK cells and heterogeneously in the immature CD56bright CD16 negative NK cell subset. NK cells expressing high amounts of TIM-3 are fully responsive with respect to cytokine production and cytotoxicity. NK cell responses may be negatively regulated when NK cells encounter target cells expressing cognate ligands of TIM-3 [36]. High levels of TIM-3 expression by total NK cells and the CD56dim effector NK cell subset in chronic HCV infection was associated with an activated phenotype polarized toward cytotoxicity [37]. NK cells in their role as early controllers of viral infections act as rheostats, regulating CD4 T cell-mediated support for the antiviral CD8 T cells [38] and contribute to immune dysfunction and viral persistence via restricting the induction of adaptive anti-viral T cell responses and abetting the development of their exhaustion. Activated NK cells cytolytically eliminate activated CD4 T cells affecting CD8 T cell function [39, 40]. Recent studies demonstrate how NK cell depletion results in enhanced antiviral T cell immunity, which triggers virus elimination [41]. NK cell receptors may orchestrate these regulatory effects [6].

DAAs not only suppress HCV replication but may also possess immunological effects. Successful IFN-free daclatasvir and asunaprevir (DCV/ASV) resulted in an improved functional NK cell response, such as NK cell degranulation and TRAIL expression to *in vitro* stimulation with IFN-α. A rapid decrease in viremia and the level of inflammatory cytokines was associated with decreased activation of intrahepatic and blood NK cells by IFN-α and was followed by the restoration of a normal NK cell phenotype and function [7]. A recent study regarding mathematical modeling suggested DAAs improve treatment response by suppressing HCV replication, which compromises the ability of HCV to induce bistability in the innate IFN signaling network, thereby lowering the fraction of cells refractory to IFN [42]. DAA via downregulating HCV core protein expression may also induce decreased regulatory T cell generation, which further promotes restoration of adaptive immune responses.

### Immune checkpoint inhibitors and hepatocellular carcinoma (HCC)

The activity regarding the checkpoint inhibitor receptors by immune cells may play a role in the maintenance of tumor tolerance. Although the role of the immune system in the pathogenesis of HCC is still unclear, various immune checkpoint inhibitor therapies combined with one another or, with sorafenib, may resuscitate exhausted T cells, affect the restoration of NK cells responses, and may lead to improved outcomes among HCC patients [43].

Direct-acting antiviral-mediated clearance of HCV is associated with the loss of intrahepatic immune activation of innate immune cells by IFN-α, which may favor decreased immune surveillance against tumor cells [44, 45]. Increased reactivations of HBV or herpesviruses observed in patients who undergo IFN-free DAA treatment also supports the DAA’s immunological effects [17, 46, 47]. The debate on whether the role of DAA on the recurrence of HCC in patients following surgery and observations of a more aggressive type of hepatocellular carcinoma than previously observed may also indicate the potential immunological effect of DAAs [48].

Since the exact immunological changes during DAA treatment in CHC patients is still unclear, in regards to this prospective study, we evaluated whether DAA treatment causes altered immune checkpoint receptor or its ligand expression by peripheral blood mononuclear cells. We investigated the phenotypic distribution of peripheral blood immune cells, their PD-1, TIM-3 and PD-L1, and Gal-9 expression prior to, at the conclusion, and 12 or 24 weeks following DAA treatment.

In our study, SVR by DAA treatment decreased the expression of inhibitory checkpoint receptors and their ligands upon innate immune cells, such as TIM-3 by immature/regulatory NKbright and NKT-like cells, PD-L1 by NK cells and Gal-9 by NK cells and monocytes. Since activated TIM-3+ NK cells cytolytically eliminate activated CD4 T cells, which affect CD8 T cell function and exhaustion [39, 40], the decrease in the percentage of activated TIM-3+ NK cells following DAA treatment may favor the re-establishment of T cell responses. DAA therapy changed TIM-3 expression differently on effector CD56dim and immature CD56bright NK subsets. Although TIM-3 expression was low regarding T cells, suppression of HCV was also associated with the significant decline of TIM-3 expression by T helper cell population. Interestingly, not only TIM-3 but its ligand Gal-9 and PD-L1 were also downregulated during post-treatment by NK cells and Gal-9 by monocytes at SVR12. The decreased Gal-9 expression on monocytes may also play a role in the regulation of NK cell activation, and via decreased TIM-3 activation, it may help to attenuate NK cell cytotoxicity and restore adaptive immune response. Further studies analyzing the changes of the immune response during successful DAA therapy in HCV infection may also help to develop new immune response based therapeutic strategies of other chronic infections, such as HBV, and may also help to decrease the risk of HCC development or recurrence following DAA therapy.

A potential weakness regarding our study is we could not obtain liver-infiltrating lymphocytes directly exposed to HCV; only peripheral blood mononuclear cells’ inhibitory checkpoint receptors and ligands expression were studied. Analyzing immune checkpoint receptor expression by intrahepatic immune cells in patients with recurrent or de novo HCC following DAA treatment may help to clear immunological mechanism, which potentially favors malignant cell proliferation and tumor progression. Another limitation of our study is we did not conduct functional tests examining NK and CD8+ cytotoxic T cells activity or cytokine production during DAA treatment. Future studies are planned to clarify the functional consequences of the observed immune checkpoint molecule expression changes. Admittedly, in view of our study, all patients suffered from advanced liver fibrosis and received dasabuvir, ombitasvir, paritaprevir/ritonavir plus ribavirin combination treatment for 12 weeks by the Hungarian National Treatment protocol [49] since, during the phase of the patient’s clinical admission, it was the only DAA drug combination reimbursed by the National Health Insurance Fund of Hungary. Although new DAA regiments, which are currently operational, contain different molecules, even without the NS3/NS4A protease inhibitor, we hypothesize the immunological effect of DAA drugs are universal, and they do not act directly upon immune cells but have an indirect effect by inducing a rapid drop of viral RNA and protein levels in the blood. Comparative studies are warranted, analyzing the same receptor expressions among patients treated with other drug combinations to confirm this hypothesis.

In the era of DAA drugs, which can eradicate HCV in nearly all individuals [50], the clinical benefit of our study offers insight regarding immune checkpoint molecules and their ligands expression changes associated with the loss of chronic antigen stimulation induced by DAA treatment. Since immune checkpoint molecules and their ligands expression are essential in tumor immunology, our data may help inch closer to the comprehensive understanding in reference to the immunological mechanisms potentially associated with early HCC emergence or recurrence and the possible causes of more aggressive HCC growth observed in some HCV cirrhosis patients following successful HCV eradication by DAAs.

## Conclusions

In conclusion, we found direct-acting antiviral treatment decreased inhibitory TIM-3, PD-L1, and TIM-3’s ligand Gal-9 expression on peripheral blood innate immune cells. Sustained virological response was associated with an increased percentage of cytotoxic T cells and decreased inhibitory TIM-3, PD-L1 receptor expression by NKT-like and immature CD56bright NK cells, and decreased Gal-9 molecule expression by monocytes. Our data suggest DAA treatment via decreasing inhibitory immune checkpoint molecules, and their ligand expression may induce the recovery of the adaptive immune responses and the functional restoration of NK cells. The potential benefit regarding this study is to clarify the immunological mechanisms associated with sustained virological response in chronic HCV infection. We believe that analyzing immunological changes associated with successful virus elimination in HCV may help to find potentially modifiable factors causing T cells exhaustion, chronicity of viral infection, and may pave a new direction in the curative treatment of other chronic viral infections, such as chronic Hepatitis B or HIV.

## Electronic supplementary material

Below is the link to the electronic supplementary material.

Supplementary material 1 (DOCX 15 kb)

## Abbreviations

HCV: Hepatitis C virus
HCC: Hepatocellular carcinoma
CHC: Chronic hepatitis C
PD-1: Programmed death-1 protein
PD-L1: Programmed death-ligand 1
TIM-3: T cell immunoglobulin and mucin-domain containing-3
Gal-9: Galectin-9
CTLA-4: The cytotoxic T-lymphocyte-associated antigen 4
HBV: Hepatitis B virus
HIV: Human immunodeficiency virus
IFN: Interferon
TNF: Tumor necrosis
BL: Baseline
EOT: End of treatment
SVR: Sustained virological response
DAA: Direct-acting antiviral
Treg: Regulatory T cell
