# Supplementary material for: Direct-acting antiviral treatment downregulates immune checkpoint inhibitor expression in patients with chronic hepatitis C
Source: Clin Exp Med. 2020 Feb 27;20(2):219–30. doi: 10.1007/s10238-020-00618-3 (PMC7181552; doi:10.1007/s10238-020-00618-3)
Supplement: Supplementary file 1 — Supplementary material 1 (DOCX 15 kb) [file 10238_2020_618_MOESM1_ESM.docx]

**Supplementary Table 1.** Peripheral blood mononuclear cells characteristics in HCV patients on DAA treatment.

|  | ***BL*** | ***EOT*** | ***SVR12*** | ***SVR24*** |
| --- | --- | --- | --- | --- |
| **CD3+ T** | 47.40±3.06 | 54.02±3.58 | **56.33±2.91^*^** | **58.36±3.22^***^** |
| **CD4+ Th** | 29.54±2.53 | 35.16±3.70 | 35.45±3.13 | 37.04±3.73 |
| **CD8+ Tc** | 12.28±1.70 | 13.43±2.00 | 14.67±1.89 | **15.28±1.59^***^** |
| **NK** | 26.29±2.72 | 21.99±2.83 | 21.40±2.42 | 21.09±2.62 |
| **NK^dim^** | 23.32±2.72 | 19.51±2.84 | 19.78±2.42 | 19.44±2.70 |
| **NK^bright^** | 3.10±0.63 | 2.58±0.46 | **1.73±0.32^*** $^** | **1.64±0.30^*** $^** |
| **NKT-like** | 5.82±1.25 | 5.18±0.97 | 5.68±1.08 | 5.56±1.04 |
| **Treg** | 2.16±0.40 | 1.69±0.15 | 2.65±0.54 | 2.08±0.22 |
| **Monocyte** | 7.05±0.47 | 6.84±0.49 | 6.54±0.44 | 5.78±0.38 |

*** p<0.01 vs. „BL” group

** p<0.03 vs. „BL” group

* p<0.05 vs. „BL” group

$ p<0.03 vs. „EOT” group
